# Birthweight trends for babies born using Assisted Reproductive Treatments between 1991 and 2018: a national cohort study

**DOI:** 10.1093/humrep/deag140

**Published:** 2026-09-25

**Authors:** Fiskani J M Kondowe, Matthew Gittins, Peter Clayton, Daniel R Brison, Stephen A Roberts

**Affiliations:** Division of Population Health, Health Services Research & Primary Care, School of Health Sciences, Faculty of Biology, Medicine and Health, Centre for Biostatistics, The University of Manchester, Manchester Academic Health Science Centre, Manchester, UK; Mathematical Sciences Department, University of Malawi, Zomba, Malawi; Division of Population Health, Health Services Research & Primary Care, School of Health Sciences, Faculty of Biology, Medicine and Health, Centre for Biostatistics, The University of Manchester, Manchester Academic Health Science Centre, Manchester, UK; Division of Developmental Biology and Medicine, Child Health & Paediatric Endocrinology, School of Medical Sciences, Faculty of Biology, Medicine and Health, The University of Manchester, Manchester Academic Health Sciences Centre, Manchester, UK; Division of Developmental Biology and Medicine, Maternal & Fetal Health Research Centre, School of Medical Sciences, Faculty of Biology, Medicine and Health, The University of Manchester, Manchester Academic Health Sciences Centre, Manchester, UK; Maternal and Fetal Health, St Mary’s Hospital, Manchester University NHS Foundation Trust, Manchester Academic Health Sciences Centre, Manchester, UK; Division of Population Health, Health Services Research & Primary Care, School of Health Sciences, Faculty of Biology, Medicine and Health, Centre for Biostatistics, The University of Manchester, Manchester Academic Health Science Centre, Manchester, UK

**Keywords:** birthweight, gestational age, trend, singleton births, ART

## Abstract

**STUDY QUESTION:**

Has the average birthweight (BW) for singletons born in the UK through ART changed between 1991 and 2018 relative to the general population?

**SUMMARY ANSWER:**

The average gestation-adjusted BW for ART singletons increased steadily between 1991 and 2012 before becoming almost static until 2018; these changes were not observed in the general UK population.

**WHAT IS KNOWN ALREADY:**

Many older studies have shown that ART babies are low BW (LBW) compared to naturally conceived babies. Some subsequent evidence suggests that the average BW for fresh and frozen transfer ART babies has increased over recent years.

**STUDY DESIGN, SIZE, DURATION:**

This cohort study utilized the UK Human Fertilisation and Embryology Authority (HFEA) dataset of 140 859 singleton ART babies born between 1991 and 2018 in the UK following both fresh and frozen embryo transfer (FET). Comparative aggregated data of 11 790 372 singleton babies born in the UK general population during the same period were obtained from NHS Digital.

**PARTICIPANTS/MATERIALS, SETTING, METHODS:**

The HFEA dataset was used to explore ART BW (raw or gestation-adjusted) and gestational age trends between 1991 and 2018. Appropriate regression models assessed the outcomes and adjusted for child sex, available treatment factors (fresh/frozen ET, embryo transfer stage (cleavage or blastocyst transfer), number of embryos transferred, ovarian stimulation, ICSI, treatment centre/clinic, and pre-implantation genetic screening of embryos) and patient factors (maternal age, previous IVF attempts, previous live births, previous pregnancies, ethnicity, country of treatment within the UK (England, Northern Ireland, Scotland, and Wales), funding source, total number of oocytes retrieved, and infertility causes (endometriosis, idiopathic, ovulatory, tubal diagnosis, and male factor)). BW, LBW, and macrosomia models were additionally adjusted for gestational age. Segmented trend analysis using linear splines was used to capture non-linear time effects for BW and gestation outcomes. For the general population data, mean BW and gestation outcomes were assessed as raw, unadjusted yearly averages.

**MAIN RESULTS AND THE ROLE OF CHANCE:**

Unadjusted raw BW trends show that ART babies were initially smaller than the general population, but over time, are becoming closer to it. Fully adjusted mean ART BW increased by ∼75 g between 1991 and 2012, and after that, it was almost static. A drop in ART raw BW starting in 2013 was concurrent with a drop in average gestation by an overall average of 2.5 days between 2012 and 2018. The mean raw BW in the general population remained almost static over the study period and consistently higher than the ART BW average, with a notable drop beginning in 2013, concurrent with a decline in average gestation. Between 1991 and 2000, and 2012 and 2018, average gestation trends were comparable in the ART and general population, with varying patterns between 2001 and 2012.

**LIMITATIONS, REASONS FOR CAUTION:**

We could only explore factors available in the dataset, possibly missing some unobserved/unrecorded covariates such as socio-economic and lifestyle factors. The ART dataset used required patient consent, leading to the exclusion of ∼40% of all cycles conducted during the study period.

**WIDER IMPLICATIONS OF THE FINDINGS:**

ART singletons getting bigger over time is reassuring, as fewer ART babies now have adverse birth outcomes such as LBW. This is desirable within the developmental origins of health and disease (DOHaD) framework, which links adverse child outcomes to later-life disease predisposition. These changes in ART singletons (not observed in the general population) might be the result of changes or improvements in ART technologies, clinic practices, and ART patient population characteristics. Therefore, there is a need to continue monitoring BW-related outcomes such as large for gestational age and macrosomia, which are more prevalent in frozen ET ART babies and, though directly associated with the observed increase in average ART BW, can be detrimental to child health.

**FUNDING:**

This study was funded by the EU H2020 Marie Sklodowska-Curie Innovative Training Networks (ITN) grant Dohartnet (H2020-MSCA-ITN-2018-812660).

**DISCLOSURES:**

The authors have no competing interests to declare.

**TRIAL REGISTRATION NUMBER:**

n/a.

## Introduction

ARTs have been used since 1978, when the first baby was born IVF in the UK. Subsequently, researchers have reported relationships between ART and outcomes such as successful implantation, live birth, and maternal and baby health outcomes, with clear evidence that ART babies have altered gestation and birthweight (BW) compared to naturally conceived (NC) babies (Maheshwari *et al.*, 2012, 2018; Zhu *et al.*, 2016; Chen and Heilbronn, 2017; Qin *et al.*, 2017; Cavoretto *et al.*, 2018; Castillo *et al.*, 2020a; Wennerholm and Bergh, 2020; Pinborg *et al.*, 2023; Wang *et al.*, 2025; Wu *et al.*, 2025a). BW has been used as a surrogate outcome for foetal growth (reflecting what happens during the peri-conception period, including ART procedures and early pregnancy) and linked to child growth and long-term health (Barker *et al.*, 1989; Martin *et al.*, 2017). Thus, the perinatal outcomes of ART babies have prompted investigation and some concern.

The last two decades have seen an evolution in ART practices, some of which are associated with treatment outcomes. Some notable worldwide changes in ART include an increase in cryopreservation of embryos, improvements in culture media and technologies for embryo selection, a shift to blastocyst stage (Day 5–6) from cleavage stage (Day 2–3) embryo transfer, increased use of ICSI to treat male infertility, and use of single embryo transfer to reduce the incidence of multiple births (Brezina *et al.*, 2012; Kushnir *et al.*, 2017; Vijay *et al.*, 2025; Wu *et al.*, 2025a,b). The population of patients receiving ART has also changed over time; ART is now more available to older patients and couples with a shorter duration of infertility, and increasingly, to those from ethnic minorities (HFEA, 2023).

Changes in ART practice over time have the potential to affect BW. Gestational age (GA) at birth is of interest in itself, as well as having a direct effect on BW. GA may also be influenced by changes in ART practice, such as blastocyst transfer, which is associated with an increased risk of pre-term birth (Castillo *et al.*, 2020a,b; Glujovsky *et al.*, 2026).

Some studies have documented BW trends after ART. Maas and others (2016) reported no temporal change in raw BW in their retrospective cohort study of 6265 fresh and 1030 frozen-embryo-transfer singletons conceived at a single US clinic between 1996 and 2013. On the contrary, a larger Nordic cohort study conducted between 1989 and 2007 reported a gradual decrease in low birthweight (LBW), preterm births (PTB), and small for gestational age (SGA) (<10th BW centile of babies with the same GA) in ART singletons. Further, LBW rates were reportedly steady between 1998 and 2007, after a drop in the earlier years (Henningsen *et al.*, 2015). In a single-site UK study, Castillo *et al.* (2019) showed that ART BW had increased by 7.8 g/year between 1991 and 2015, totalling an average of 180 g over 25 years. However, it is unclear whether these trends are specific to the wider UK ART population or whether similar trends are also observed in the NC population in the UK.

With the gradual advancements in ART, monitoring changes in BW trends is crucial. Additionally, identifying specific factors linked to these changes may be necessary in understanding ART’s impact on the periconceptional programming of long-term health outcomes (Brison *et al.*, 2013; Fleming *et al.*, 2018) in line with the Developmental Origins of Health and Disease (DOHaD) hypothesis (Barker *et al.*, 1989; Barker, 2007).

This study thus explored changes in BW and gestation over time in the national UK ART cohort. A parallel analysis of the full UK population of babies born during the same period was conducted for comparison.

## Materials and methods

### Datasets

We utilized data on ART cycles conducted in the UK between 1991 and 2018 (the latest data available) from the Human Fertilisation and Embryology Authority (HFEA) ART register. The HFEA ART register holds comprehensive records of all patients who have used ART since August 1991, when it became compulsory for every registered UK fertility clinic to report treatment details to the HFEA. We obtained an anonymized dataset of all own-gamete ART cycles conducted in the UK between 1991 and 2018, but including only cycles in which patients gave Consent to Disclosure (CD) of their identifying information for use in non-contact research. Consent was deemed necessary due to the low risk of patient re-identification from the supplied data. We thus accessed almost all cycles conducted before September 2009 (pre-2009) as CD rates were high (92%), but only 55% of the cycles conducted from October 2009 to 2018 gave CD (Kondowe *et al.*, 2025). For this work, we selected cycles resulting in live-born singletons, defined as one live birth from one foetal heart detected at an early pregnancy scan. We excluded singleton babies with missing BW and embryo transfer type (fresh or frozen) data. We also excluded those with missing gestation and child sex, as these factors were necessary to calculate adjusted BW. Details of the numbers and exclusions are shown in Fig. 1.

**Figure 1.**
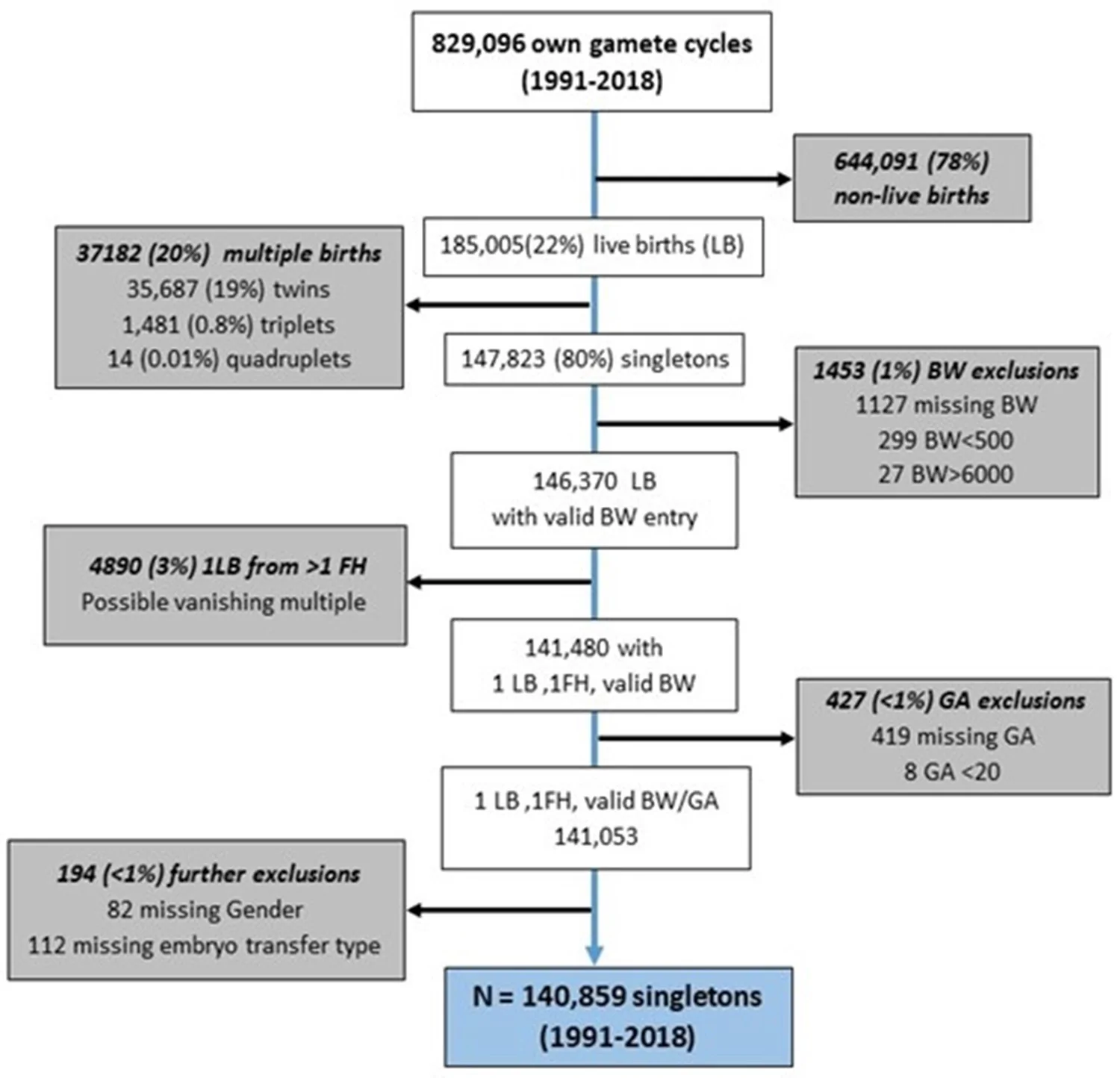
**Inclusion–exclusion criteria for the analysis subset for singleton ART babies.** Data on singleton babies born from ART cycles conducted between 1991 and 2018 in the UK. LB, live birth; FH, foetal heart; BW, birthweight; GA, gestation age.

We obtained aggregated annual population data on BW and gestation from NHS Digital. This dataset was provided as aggregated yearly counts, means, and SDs of BW and gestation for 12 302 732 singleton babies born in the UK between 1991 and 2018, aggregated by child sex (male/female) and maternal age groups (under 18, 18–34, 35–36, 37–38, 39–40, 41–44, and over 44 years). Additionally, counts were provided for babies with LBW or PTB (<37 weeks’ gestation), respectively. We excluded babies with unknown child sex or maternal age listed as ‘under 18’, leaving a total of 11 790 372 live-born singletons. This general population will include ART as well as NC singletons; however, the proportion of ART births was ∼2% over the study period, although it may reach 5% if Medically Assisted Reproduction births are included. Therefore, this is a predominantly NC population.

Ethical approval was obtained through the UK NHS Health Research Authority [20/PR/0505] and HFEA Register Research Panel [HFEA_2019_017].

### Outcome variables

The primary outcomes were raw and gestation-adjusted BW (gaBW) and GA. gaBW was estimated by including a cubic function for gestation (terms in gestation, gestation^2^ and gestation^3^) in the BW linear regression model and computing the fitted BW for the mean gestation (39.2 weeks). As individual-level data were unavailable, we were unable to compute a gestational-adjusted BW for the population data. GA (in weeks) in the ART cohort was calculated as [(Delivery date − embryo transfer date) + 14 days + days of embryo in culture]/7. We imputed missing calculated gestation (n = 946; <0.01%) using reported gestation, which was also available in the dataset.

Secondary outcomes were: LBW (BW <2500 g), high birthweight (HBW; BW >4000 g), PTB (Gestation <37 weeks), SGA (BW *Z*-score < −1.28 or BW below the 10th adjusted BW centile), and large for gestational age (LGA; BW *Z*-score >1.28 or BW above the 90th adjusted BW centile). The BW *Z*-scores were generated from WHO reference data (Kiserud *et al.*, 2017). We used full weeks gestation to calculate the *Z*-scores as recommended.

### Data analysis

Descriptive statistics are presented as counts (percentages) or mean values (±SD). Temporal BW and gestation trends in the ART dataset were assessed using linear regression models (unadjusted and adjusted for potential confounders) with embryo transfer year as the primary explanatory variable. Logistic regression was used to explore the outcomes of LBW, HBW, PTB, SGA, and LGA. The models were adjusted for child sex, available treatment factors (fresh/frozen ET, embryo transfer stage (cleavage or blastocyst transfer), number of embryos transferred (1, 2, >2), ICSI, treatment centre/clinic, and pre-implantation genetic screening of embryos (PGT)), and patient factors (maternal age in years, previous IVF attempts (none to 5+), previous live births (none to 2+), previous pregnancies (none to 3+), ethnicity, country of treatment within the UK (England, Northern Ireland, Scotland, and Wales)), funding source, total number of oocytes retrieved, and infertility causes (endometriosis, idiopathic, ovulatory, tubal diagnosis, and male factor). For visualization, analogous models were fitted using a categorical variable for year, thus giving fitted estimates for each year.

As a simple linear time trend did not capture important non-linear effects, a segmented function was fitted to estimate trends in gestation and BW over different time periods. Linear splines were used for BW (1991–2006, 2006–2012, and 2012–2018) and gestation (1991–1997, 1997–2012, and 2012–2018). The split points were determined through exploratory analyses using an incremental method, assessing model fit using the Akaike Information Criterion at various cut points. We further explored interactions between time and child sex, ovarian stimulation, fresh or frozen embryos, and embryo transfer stage.

In a separate exploratory analysis using population data, we plotted raw unadjusted BW (mean BW) and gestation (mean gestation) by year, comparing observed trends to the HFEA ART trends during the study period.

For the primary outcomes in the ART group, with and without covariate adjustment, we applied previously derived weights (Kondowe *et al.*, 2025) to correct for the loss of patients due to the absence of Consent to Disclosure (CD).

All data manipulation and statistical analyses were performed using Stata (versions 17 and 18; StataCorp, College Station, TX, USA), and 95% CIs for the estimates are presented.

## Results

### Study population and characteristics

The final ART dataset included 140 859 live-born singletons from cycles conducted between 1991 and 2018, of which 112 530 (80%) were born following fresh embryo transfers and 28 329 (20%) following frozen embryo transfers (FETs). Summaries for maternal and treatment factors and child outcomes are presented in Table 1.

**Table 1.** Treatment, parental, and child characteristics in the ART cohort.

*Maternal/patient factors*			*Treatment factors*		
**Maternal age**			**Embryo transfer type**		
Mean (SD)	33.7	(4.1)	Fresh	112 530	(79.9)
**Ethnicity**			Frozen	28 329	(20.1)
White	55 287	(39.2)	**Embryo transfer stage**		
Asian	6175	(4.4)	Cleavage	88 546	(62.9)
Black	1210	(0.9)	Blastocyst	52 313	(37.1)
Mixed	997	(0.7)	**Ovarian stimulation used**		
Other	1711	(1.2)	Yes	112 389	(79.8)
Not stated	75 479	(53.6)	(Missing)	10	(0.0)
**Previous IVF treatments**			**ICSI**		
None	66 680	(47.3)	Yes	61 881	(43.9)
1	35 858	(25.5)	(Missing)	14 620	(10.4)
2	18 777	(13.3)	**Embryo biopsy**		
3	9378	(6.7)	PGT-A	804	(0.6)
4	4754	(3.4)	PGT-M	1235	(0.9)
5+	5412	(3.8)	**Number of eggs collected**		
**Previous live births**			Mean (SD)	8.9	(6.8)
0	123 620	(87.8)	**Number of Embryos transferred**		
1	16 470	(11.7)	1	48 500	(34.4)
2+	769	(0.5)	2	75 816	(53.8)
**Previous pregnancies**			3+	16 378	(11.6)
0	118 641	(84.2)	(Missing)	165	(0.1)
1	19 976	(14.2)	***Child outcomes***		
2	2016	(1.4)	**Birthweight (g)**		
3+	226	(0.2)	Mean (SD)	3294.0	(605.5)
**Funding source**			**Gestation (weeks)**		
NHS Funded	37 527	(26.6)	Mean (SD)	39.2	(2.2)
Private	41 851	(29.7)	**Dichotomized outcomes**		
Not Recorded	61 481	(43.6)	LBW	11 395	(8.1)
**Infertility causes**			HBW	13 032	(9.3)
Male factor	65 218	(46.3)	PTB	12 775	(9.1)
Tubal factor	28 148	(20.0)	SGA	24 976	(17.7)
Ovulatory	19 215	(13.6)	LGA	7 398	(5.3)
Endometriosis	10 165	(7.2)	**Sex**		
Unknown	43 361	(30.8)	Female	69 175	(49.1)
			Male	71 684	(50.9)

Based on all 140 859 patients in the cohort. N (%) or mean (SD) as indicated.

PGT-M, pre-implantation genetic testing of an embryo for known hereditary genetic defects; PGT-A, pre-implantation genetic testing for chromosomal defects; SGA, small for gestational age; LGA, large for gestational age; LBW, low birthweight; HBW, high birthweight (macrosomia); PTB, preterm birth.

Over the cohort period, there were substantial changes in patient and treatment characteristics (see Supplementary Fig. S1), including increasing patient age, a shift from cleavage to blastocyst transfer, increased use of single-embryo transfer, the virtual elimination of triple-embryo transfer, and increased use of ICSI and frozen-embryo transfer.

### BW and gestation outcome trends

Figure 2 shows the trends over time for the three primary outcomes. The raw BW shows increases till around 2012, then decreases as the gestation decreases. After adjusting for patient and treatment characteristics—that is, comparing patients who had similar treatments, there was an upward trend between 1991 and 2006, with a steep decline after 2012, corresponding to a decline in gestation over the same period. gaBW shows a steady increase over the whole period in both adjusted and unadjusted models.

**Figure 2.**
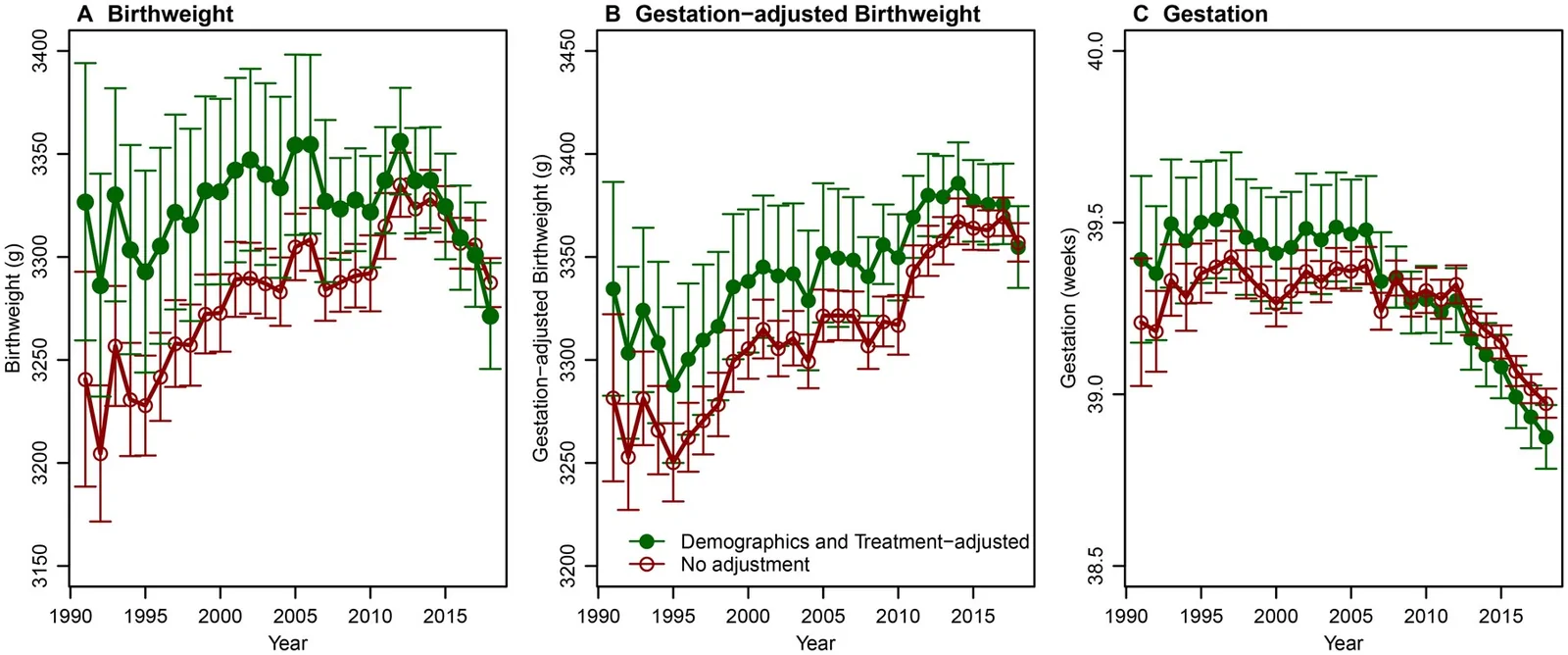
**Birthweight and gestation over time. **(**A, B**) birthweight; (**C**) gestation.  Adjusted models include child sex, embryo transfer type and stage, ICSI, ovarian stimulation, maternal age, number of previous pregnancies, live births and IVF treatments, number of oocytes collected, ethnicity, funding, infertility causes, clinic/treatment centre, PGT, and number of embryos transferred. Gestation adjustment uses a cubic function. PGT, pre-implantation genetic screening of embryos.

Overall, we see an increase in adjusted raw BW of ∼3–7g/year (∼82 g over 1991–2012), a trend which continues for adjusted gaBW (∼75 g between 1991 and 2012). However, adjusted raw BW declines by ∼11 g/year after around 2012, as the adjusted gestation declines by 0.45 days/year (Table 2).

**Table 2.** Model estimates of trends in gestation-adjusted birthweight (gaBW), unadjusted BW (rawBW), and gestation.

	Patient and treatment adjustment		**Segmented trend^b^**		
		Linear trend	1991–2006	2006–2012	2012–2018
		(g/year [95% CI])	(g/year [95% CI])		

**RawBW**	Unadjusted	2.7	4.4	4.8	−4.3
		[2.2, 3.1]	[3.3, 5.6]	[2.8, 6.8]	[−6.5, −2.1]
	Adjusted	−0.8	2.8	6.8	−11.3
		[−1.9, 0.3]	[1.3, 4.3]	[2.9, 10.7]	[−13.7, −8.9]

			**1991–2006**	**2006–2012**	**2012–2018**

**gaBW^a^**	Unadjusted	4.3	3.8	5.6	3.1
		[4.0, 4.6]	[3.0, 4.7]	[4.1, 7.2]	[1.4, 4.8]
	Adjusted	2.4	3.0	9.9	−2.8
		[1.6, 3.3]	[1.9, 4.2]	[6.9, 12.9]	[−4.7, −0.9]

			**1991–1997**	**1997–2012**	**2012–2018**

		(days/year [95% CI])	(days/year [95% CI])		

**Gestation**	Unadjusted	−0.09	0.14	−0.03	−0.38
		[−0.11, −0.08]	[0.03, 0.25]	[−0.05, −0.01]	[−0.43, −0.33]
	Adjusted	−0.16	0.10	−0.07	−0.45
		[−0.19, −0.14]	[−0.02, 0.22]	[−0.11, −0.02]	[−0.50, −0.39]

All ‘adjusted’ models are regression (linear and segmented) models adjusted for child sex, embryo transfer type and stage, ICSI, ovarian stimulation, maternal age, number of previous pregnancies, live births and IVF treatments, number of oocytes collected, PGT, ethnicity, number of embryos transferred, funding source, infertility causes, and clinic/treatment centre.

a Model adjusted for gestation (as a cubic function).

b Year modelled as a split function for birthweight (linear splines: 1991–2006, 2006–2012, and 2012–2018) and gestation (linear splines: 1991–1997, 1997–2012, and 2012–2018) models.

Trends in LBW, HBW, PTB, SGA, and LGA follow as would be expected from the changes in mean BW and are shown in Supplementary Fig. S2. Notably, there has been a decrease in clinically small babies (adjusted SGA decreased from around 16 to 11% over the period, LBW from 11 to 8%). Conversely, LGA rates increased from 3.5 to 5.5% over the same period, although the HBW rate decreased as the gestation decreased following an initial increase.

### Factors associated with BW and gestation

The full fitted models are available in Supplementary Table S1. The main factors associated with BW are the baby’s sex, maternal pregnancy history, and whether a fresh or frozen embryo was transferred. Frozen embryos were associated with an 80 [45, 114] g increase in gaBW, comparable to the 135 [130.6, 140.2] g difference between the sexes and the 81 [66.5, 94.9] g difference between first and subsequent pregnancies. The associations of the other factors included in the model are small (Supplementary Table S1) but do include a small notable effect where blastocyst transfers have a shorter gestation (−0.04 [−0.08–0.00] weeks and a corresponding increase in gaBW (11.4 [3.3, 19.5] g). Gestation is associated with pregnancy history and sex but only weakly associated with treatment characteristics.

Over the period for this cohort, the gaBW increased somewhat faster in fresh transfers than in FETs, and the difference between the two modalities appears to have decreased over time (Fig. 3). However, a formal interaction test did not reach statistical significance (*P* = 0.082), and given the number of possible interactions tested, this remains an unproven observation. No other patient or treatment variables showed any differences in BW trend.

**Figure 3.**
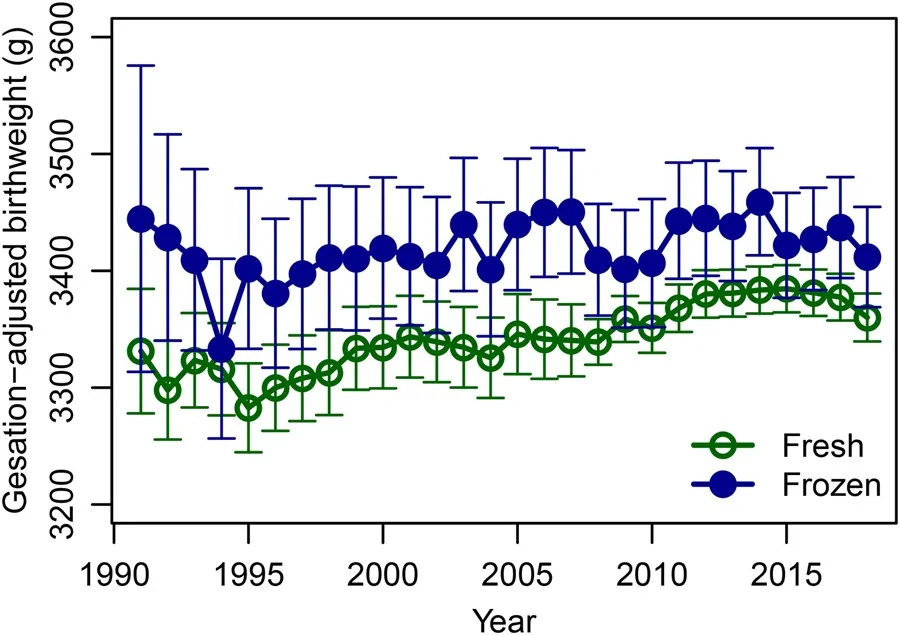
**Gestation-adjusted BW over time for fresh and frozen embryo transfers.** Model adjusted for child sex, embryo transfer type and stage, ICSI, Ovarian stimulation, maternal age, number of previous pregnancies, live births and IVF treatments, number of oocytes collected, PGT, ethnicity, number of embryos transferred, funding source, infertility causes and clinic/treatment centre. BW, birthweight; PGT, pre-implantation genetic screening of embryos.

### Correction for non-consent

The dataset used was subject to *Patient CD*, excluding ∼40% of all cycles conducted during the study period (Kondowe *et al.*, 2025). We applied weights estimated in Kondowe *et al.* (2025) to adjust for the excluded cycles, and the results showed that the BW and gestation trends did not change appreciably after weighting (Supplementary Fig. S3).

### BW and gestation trends in the general population

Plots of raw BW trends showed that, on average, ART babies were initially smaller but have been catching up to population BW averages in recent years (Fig. 4A). We note that the absolute values in Fig. 4 are not directly comparable, as there is no adjustment for maternal characteristics which differ between the two groups. The population mean BW has remained nearly static over time and has consistently been higher than the ART BW average. There was a notable drop in average BW in both cohorts starting in 2012, concurrent with a decline in average gestation beginning the same year. Average gestation trends were comparable in ART and population pregnancies between 1991 and 2000 and between 2012 and 2018, with varying patterns between 2001 and 2012 (Fig. 4B).

**Figure 4.**
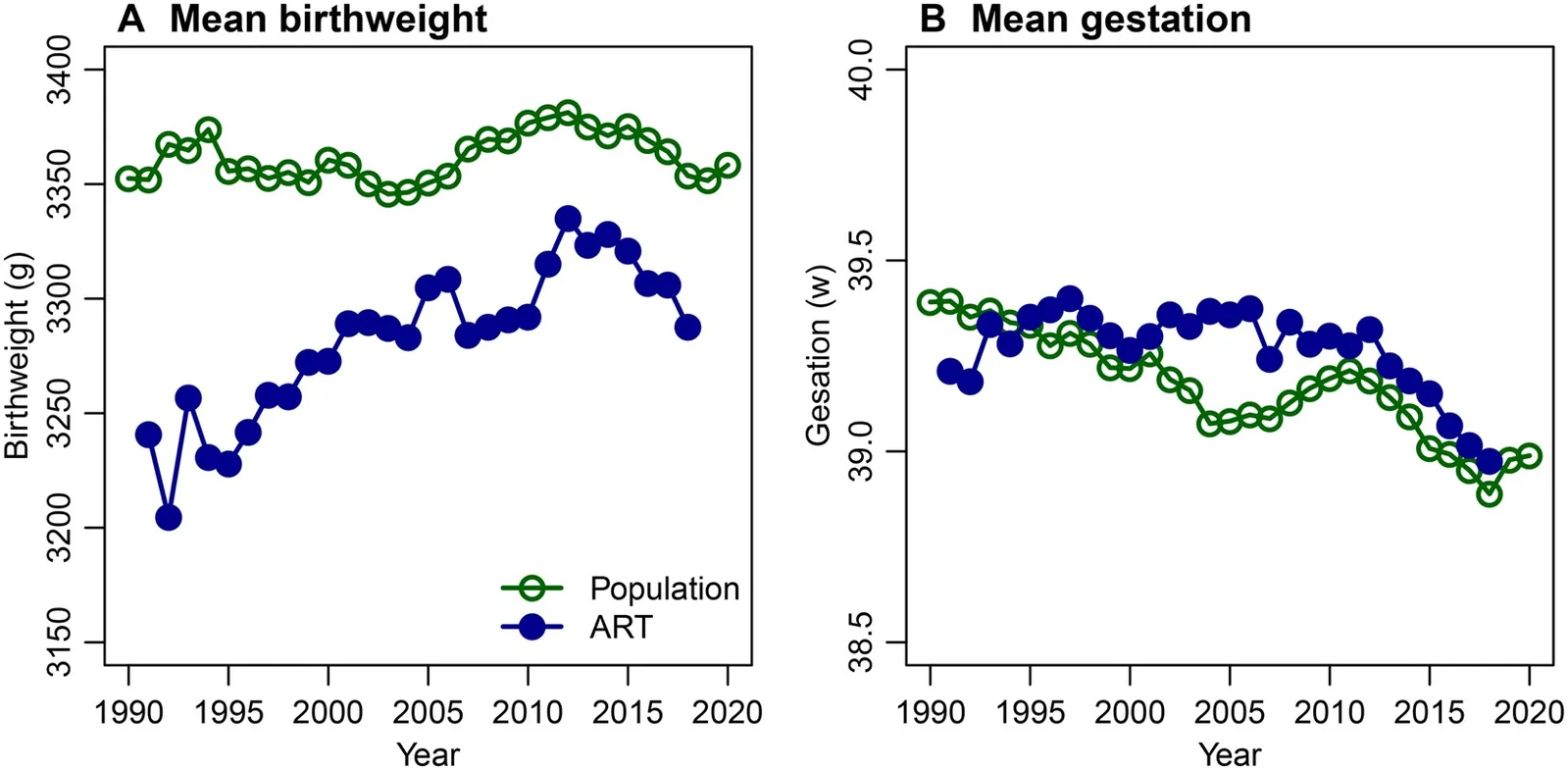
**Unadjusted average BW and gestation trends in the ART vs UK General population births. **(**A**) BW and (**B**) gestation length. Mean BW and gestation per year. BW, birthweight.

## Discussion

This is one of the few large studies exploring population-level trends in singleton ART BW over time. The results show that when adjusted for available patient and treatment factors, the average adjusted gestation-adjusted ART BW in the UK increased by ∼75 g between 1991 and 2012. After 2012, the trend was nearly constant in the adjusted models, with a marginally significant decrease. Notably, the 2013 drop in average BW in the current study coincided with the onset of a significant negative trend in average gestation. Two notable underlying trends were observed throughout the study period: an increase in large babies (LGA) and a decrease in small babies (LBW and SGA).

These results confirm those from a UK single-centre study by Castillo *et al.* (2019), reporting a historical (1991–2015) increase in average BW (by 7.4 g/year) in the ART population. Other groups have explored perinatal outcome trends in ART babies and report a general reduction in adverse outcomes over time (Henningsen *et al.*, 2015; Maas *et al.*, 2016). Henningsen *et al.*’s (2015) Nordic register study reported a gradual decrease in PTB, LBW, and SGA rates between 1989 and 2007, changes that were coupled with an increase in IVF treatment utilization. In their single-centre USA study of 6265 fresh and 1030 frozen embryo singletons, Maas *et al.* (2016) found no temporal change in unadjusted BW between 1996 and 2013. However, that study did not adjust for important factors (including gestation); thus, it is not comparable with adjusted BW results.

The UK national-level population data revealed that unadjusted singleton BW remained consistently higher than ART singleton BW; the trend was almost steady, with only a slight decrease (by 14.7 g) in average BW, from 3333 g in 1991 to 3318.3 g in 2018. Alberman and Botting (1991) reported that BW increased in the general population throughout the 1970s–1980s, yet this trend did not appear to have continued after the 1990s (Bonellie *et al.*, 2008). Other cohort studies have shown very small or no BW trends over the past decades in the general population in France (Diouf *et al.*, 2011), Germany (Schiessl *et al.*, 2009), China (Guo *et al.*, 2014), and the USA (Gibson *et al.*, 2015).

The results from our study are reassuring, as they imply fewer ART babies now have adverse birth outcomes such as LBW and SGA, which is desirable within the DOHaD framework that links restricted foetal growth to later-life disease predisposition. However, this trend also represents a shift in the BW distribution, with LGA increasing from 3.5 to 5.5%. This suggests a potential trade-off: while fewer ART singletons are now born small, a growing proportion are born large. Within the DoHad framework, LGA and high BW are associated with later obesity and metabolic risk, although long-term evidence among ART-conceived offspring is still developing. As such, there is also a need to monitor extreme BW-related outcomes such as LGA and HBW, which are more prevalent in babies born from frozen embryos and, though directly associated with increased average BW, may translate into differences in childhood and adult cardiometabolic health.

Interestingly, the current study observed similar trends in gestation in both the ART and general population cohorts, showing a steep drop after 2012. This may be related to changes in hospital pregnancy management policies and practices, especially increased rates of labour induction and caesarean delivery (Henningsen *et al.*, 2015; Widdows *et al.*, 2021). The 2012 drop in gestation could reflect trends in caesarean section rates (including both emergency and elective), which have almost doubled to 34% in 2021 from 18% in the late 90s (NHS Digital, 2021). These changes may suggest that more mothers who would have delivered at a later gestation (>39 weeks) are delivering slightly earlier. The NHS England ‘Saving Babies Lives’ initiative, formally launched in 2015, was associated with an increase in inductions and caesarean sections (Widdows *et al.*, 2021) and would account, at least in part, for the reduction in gestation over the later period, particularly as this trend in the data is driven by a decrease in gestations >40 weeks and over-large babies (Supplementary Fig. S4). Within the ART population, the drop in gestation can be associated with similar changes in general management and care for ART pregnancies, coupled with professionals possibly becoming more cautious and aware of avoiding the potential complications related to ART, thus treating ART pregnancies as high-risk and further encouraging early and monitored deliveries. A particularly strong decrease in the proportion of babies born beyond 42 weeks was observed around 2012 among the ART population, further suggesting increased interventions for overdue babies (Supplementary Fig. S4).

As the results and literature show minimal or no increase in BW in the general population, the increase in BW (with or without adjustment for gestation) in the ART population appears specific to ART. Although the specific causes are unknown, this increase in BW could be linked to changes in patient demographics of couples seeking ART and continued improvements in clinical and laboratory technologies. For instance, we observed a general increase in average maternal age and the proportion of ethnic minorities (Supplementary Fig. S1) seeking ART in the UK, both of which are associated not only with child outcomes but may also serve as surrogates for unavailable population factors such as socio-economic status. Over this period, there has been a general increase in the number of ART treatments (from ∼1% of births at the start of the period to ∼3% currently), and this will have resulted in changes to the socioeconomic characteristics of the recipient populations, which are not captured in the registry data.

There is a debate regarding the impact of add-ons on ART outcomes. Much focus has been placed on their impact on successful live births, while fewer studies have examined child outcomes. We included pre-implantation genetic testing for aneuploidy (PGT-A) and for monogenic disorders in the analysis. The results did not show any major differences in outcomes between babies born after PGT and those not, but babies born after PGT-A were slightly larger by 27.9 [0.5–55.2] g. Despite the data lacking in comprehensiveness, this is one of the largest samples of PGT offspring. These results were consistent with those published in previous studies (Hou *et al.*, 2021; Zheng *et al.*, 2021; Alteri *et al.*, 2023; Belva *et al.*, 2023; Ginström Ernstad *et al.*, 2023).

There have been many improvements in embryo laboratory procedures, including changes in embryo culture media and techniques; however, in this registry study, we were unable to explore many laboratory factors as they were not available in the dataset. Castillo *et al.* (2019) mapped changes in embryo culture conditions to average BW trends in a single clinic site between 1991 and 2014. We explored and controlled for some of the mapped factors in Castillo *et al.*’s (2019) study, such as the introduction of ICSI, increased use of single-embryo transfer, and shifts toward more blastocyst and frozen-embryo transfers, all of which may influence foetal growth and BW. Historically, FET’s have been associated with higher mean BW and lower risks of LBW and small-for-gestational-age birth compared with fresh transfers. As these effects were controlled for in the analysis, they do not explain the changes in both adjusted gaBW and raw BW. The most recent Cochrane review confirms an association between blastocyst transfer and PTB (Glujovsky *et al.*, 2026), and the models here also showed a small effect of transfer stage on gestation and gaBW (Supplementary Table S1). Castillo’s study further reported increasingly optimized embryo culture and incubation systems over time. Since the ART laboratory serves as a substitute maternal environment before the embryo is introduced into the mother’s womb, these improved systems, which are widely implemented across the UK, may impact national-level ART outcomes. It is noteworthy that the discussed mechanisms are unlikely to act independently, and the observed increase in BW probably reflects a combination of evolving ART technologies, clinic policies, and patient characteristics, including factors that are not captured in the HFEA register, rather than a single causal factor.

### Limitations

The study’s major limitations were: (i) the unavailability of comprehensive data on ART treatment laboratory factors and some important confounders, such as maternal smoking, weight, BMI, and socio-economic status, and (ii) the inability to directly compare the ART and general population outcomes since the available population data was only available in aggregated form and the mode of conception was not recorded. However, we were still able to explore important ART treatment factors in the dataset and reported notable trends. (iii) The dataset used was subject to Patient CD, excluding ∼40% of all cycles conducted during the study period. We applied weights estimated in related work (Kondowe *et al.*, 2025) to adjust for the excluded non-CD cycles, and the results showed that the general BW and gestation trends did not change appreciably after weighting the data to allow for non-consent. However, bias due to unmeasured confounders of consent cannot be ruled out. Further exploration of more complex weighting procedures, including treatment parameters, did not materially change this conclusion (Kondowe *et al.*, 2023). Although the inclusion of treatment modalities in the weighting model did make a small difference to the estimates, this did not materially affect the conclusions, and such an additional adjustment based on a post-consent variable is not generally advised. Nevertheless, given the potential for bias, it was essential that the issue of non-consent was considered, and we were able to demonstrate that, unlike previous studies of treatment success rates (Kondowe *et al.*, 2025, Non-CD was unlikely to be a major issue here.

## Conclusion

Using a large national-level dataset, we have shown that ART singletons are generally getting heavier over time, yet this increase in BW is not observed in the general population. The observed ART trends may be attributable to changes in ART technologies, clinic practices, and patient characteristics. To our knowledge, this is one of the largest national-level studies reporting ART BW and gestation trends and the largest in the UK ART population.

## Supplementary Material

deag140_Supplementary_Figure_S1

deag140_Supplementary_Figure_S2

deag140_Supplementary_Figure_S3

deag140_Supplementary_Figure_S4

deag140_Supplementary_Table_S1

## Data Availability

The ART dataset was provided by the HFEA under a data sharing agreement and cannot be distributed further by the authors. However, the data can be obtained directly from the HFEA (https://www.hfea.gov.uk/about-us/data-research/). Similarly, the population data can be obtained from NHS Digital reference: NIC-571004-P4H3F.
