# Supplementary material for: Birthweight trends for babies born using Assisted Reproductive Treatments between 1991 and 2018: a national cohort study
Source: Hum Reprod. 2026 Sep 25;41(11):1984–92. doi: 10.1093/humrep/deag140 (PMC13726750; doi:10.1093/humrep/deag140)
Supplement: Supplementary file 2 [file deag140_supplementary_figure_s2.pdf]

*Human Reproduction*, 2026, 1–1
https://doi.org/10.1093/humrep/deag140
**Supplementary Data**

human reproduction
OXFORD

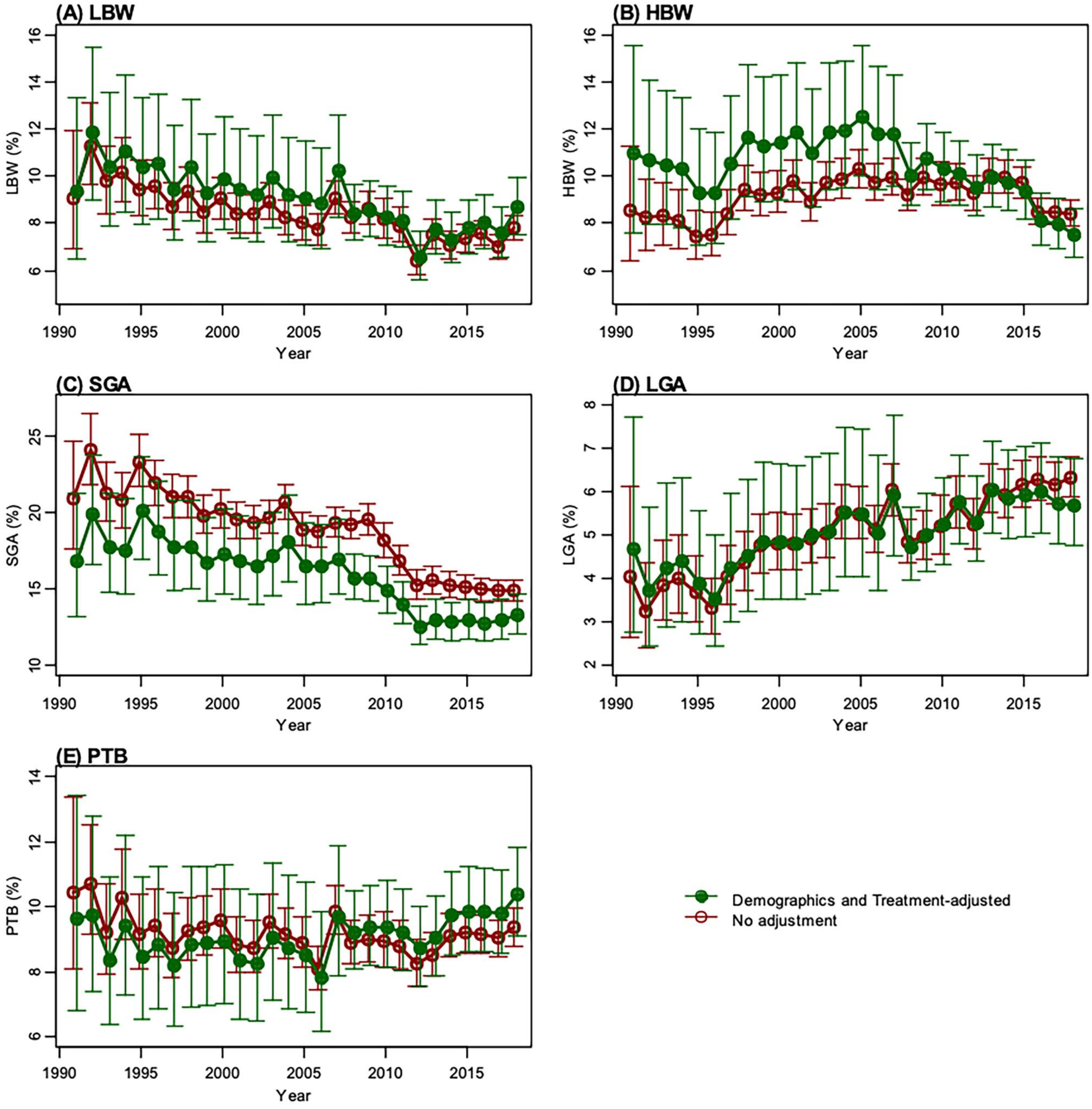


**Supplementary Figure S2.** **(A) Low birthweight (LBW), (B) High birthweight (HBW), (C) Small for gestational age (SGA), (D) Large for gestational age (LGA), and (E) pre-term birth (PTB) trends.** Proportions are based on logistic regression models adjusted for child sex, embryo transfer type and stage, number of embryos transferred, ICSI, ovarian stimulation, maternal age, number of previous live births and IVF treatments, number of oocytes collected, PGT, ethnicity, funding source, infertility causes and clinic/treatment centre. PGT, pre-implantation genetic screening of embryos.
