# Supplementary material for: Birthweight trends for babies born using Assisted Reproductive Treatments between 1991 and 2018: a national cohort study
Source: Hum Reprod. 2026 Sep 25;41(11):1984–92. doi: 10.1093/humrep/deag140 (PMC13726750; doi:10.1093/humrep/deag140)
Supplement: Supplementary file 3 [file deag140_supplementary_figure_s3.pdf]

Human Reproduction, 2026, 1–1
https://doi.org/10.1093/humrep/deag140
Supplementary Data

human reproduction
OXFORD

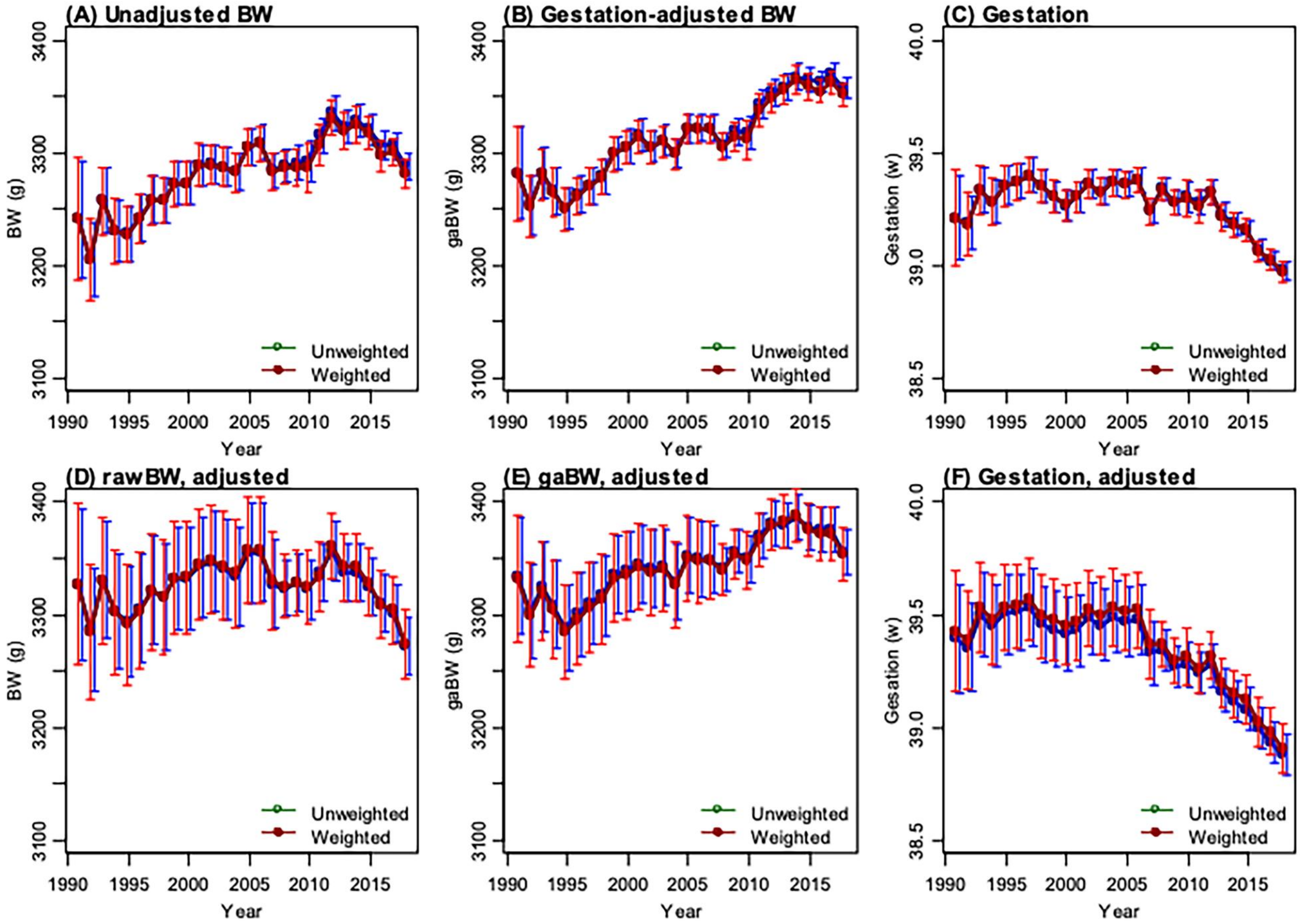

Supplementary Figure S3. Adjustment for non-consent. Unadjusted BW, gestation-adjusted BW (gaBW), and gestation models with and without reweighting to correct for non-consent. Upper panels (**Figures A**, **B**, **C**) are not adjusted for patient and treatment characteristics, lower panes (**Figures D**, **E**, **F**) were adjusted for sex, embryo transfer type and stage, number of embryos transferred, ICSI, ovarian stimulation, maternal age, number of previous pregnancies, live births and IVF treatments, number of oocytes collected, ethnicity, funding source, PGT, infertility causes, and clinic/treatment centre. Points offset slightly for clarity. BW, birthweight; PGT, pre-implantation genetic screening of embryos.
