# Supplementary material for: Birthweight trends for babies born using Assisted Reproductive Treatments between 1991 and 2018: a national cohort study
Source: Hum Reprod. 2026 Sep 25;41(11):1984–92. doi: 10.1093/humrep/deag140 (PMC13726750; doi:10.1093/humrep/deag140)
Supplement: Supplementary file 4 [file deag140_supplementary_figure_s4.pdf]

Human Reproduction, 2026, 1–1
https://doi.org/10.1093/humrep/deag140
Supplementary Data

human reproduction
OXFORD

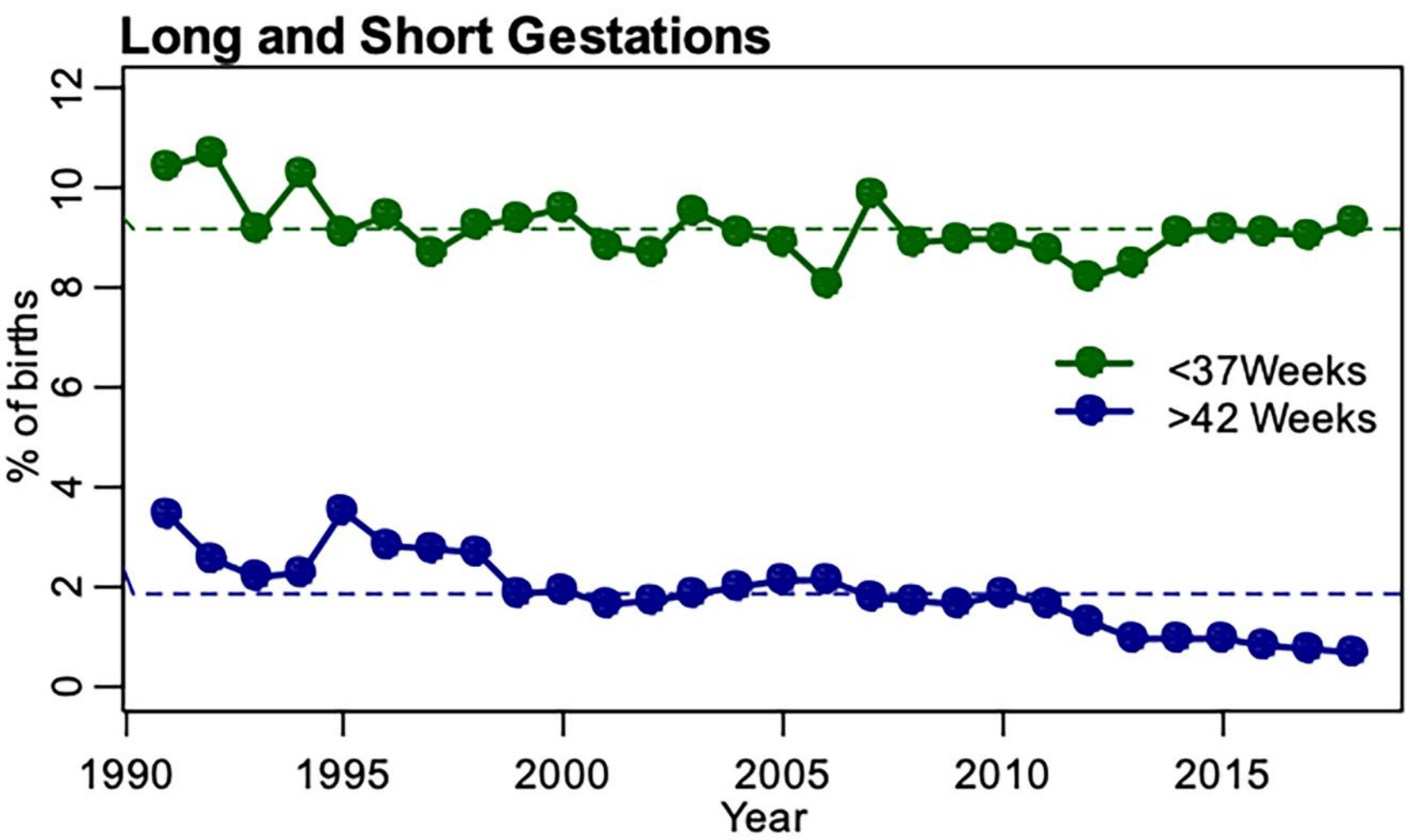

Supplementary Figure S4. Trends of proportions in different gestation bands in the ART cohort. Broken lines show the mean proportions.
