# Supplementary material for: Birthweight trends for babies born using Assisted Reproductive Treatments between 1991 and 2018: a national cohort study
Source: Hum Reprod. 2026 Sep 25;41(11):1984–92. doi: 10.1093/humrep/deag140 (PMC13726750; doi:10.1093/humrep/deag140)
Supplement: Supplementary file 5 [file deag140_supplementary_table_s1.pdf]

Human Reproduction, 2026, 1–2
https://doi.org/10.1093/humrep/deag140
Supplementary Data

human reproduction OXFORD

Supplementary Table S1. Full fitted models.

		Raw BW (g)	gaBW (g)[1]	Gestation (weeks)
**Sex**	Female	Ref	Ref	Ref
	Male	109.5 [103.3, 115.7]	135.4 [130.6, 140.2]	−0.2 [−0.2, −0.1]
**Transfer type**	Fresh	Ref	Ref	Ref
	Frozen	77.9 [33.0, 122.7]	79.5 [44.9, 114.1]	0.04 [−0.12, 0.20]
**Transfer stage**	Cleavage	Ref	Ref	Ref
	Blastocyst	3.5 [−7.0, 13.9]	11.4 [3.3, 19.5]	−0.04 [−0.08, 0.00]
**ICSI**	IVF	Ref	Ref	Ref
	ICSI	16.5 [8.2, 24.8]	3.1 [−3.3, 9.6]	0.08 [0.05, 0.11]
	Missing	12.8 [−2.8, 28.3]	5.2 [−6.8, 17.2]	0.04 [−0.02, 0.09]
**Stimulation used**	No	Ref	Ref	Ref
	Yes	−42.1 [−86.2, 2.2]	−8.1 [−42.3, 26.0]	−0.19 [−0.35, −0.03]
	Missing	301.9 [−68.9, 672.6]	206.4 [−79.3, 492.1]	0.50 [−0.84, 1.83]
**PGT_A**	No	Ref	Ref	Ref
	Yes	13.7 [−21.8, 49.2]	27.9 [0.5, 55.2]	−0.08 [−0.21, 0.04]
**PGT_M**	No	Ref	Ref	Ref
	Yes	12.0 [−31.4, 55.4]	3.4 [−30.0, 36.8]	0.02 [−0.13, 0.18]
**Maternal age**				
**Linear term**	Per year	31.1 [21.8, 40.5]	7.3 [0.1, 14.5]	0.17 [0.13, 0.20]
**Quadratic term**	Per year	−0.5 [−0.6, −0.3]	−0.1 [−0.2, 0.0]	−0.00 [−0.00, −0.00]
**Eggs collected**	Per egg	−1.7 [−2.4, −1.1]	−1.0 [−1.4, −0.5]	−0.00 [−0.01, −0.00]
**Ethnicity**	White	Ref	Ref	Ref
	Asian	−235.7 [−251.6, −219.7]	−177.9 [−190.2, −165.6]	−0.36 [−0.42, −0.31]
	Black	−198.1 [−232.2, −164.0]	−92.7 [−119.0, −66.5]	−0.60 [−0.72, −0.48]
	Mixed	−116.4 [−153.7, −79.1]	−90.4 [−119.1, −61.7]	−0.15 [−0.29, −0.02]
	Other	−88.9 [−117.8, −60.1]	−61.3 [−83.5, −39.1]	−0.16 [−0.26, −0.06]
	Not stated	−40.1 [−52.3, −27.8]	−29.1 [−38.6, −19.7]	−0.07 [−0.11, −0.02]
**Previous IVF cycles**	0	Ref	Ref	Ref
	1	8.2 [−0.5, 16.9]	5.9 [−0.7, 12.6]	0.02 [−0.01, 0.05]
	2	1.7 [−9.1, 12.5]	0.4 [−7.9, 8.8]	0.01 [−0.03, 0.05]
	3	−2.8 [−16.9, 11.3]	−3.9 [−14.8, 7.0]	0.00 [−0.05, 0.05]
	4	−22.8 [−41.45, −4.1]	−3.1 [−17.6, 11.3]	−0.11 [−0.18, −0.04]
	5+	−9.2 [−27.8, 9.3]	13.8 [−0.5, 28.1]	−0.13 [−0.20, −0.07]
**Previous IVF pregnancies**	0	Ref	ref	Ref
	1	−20.0 [−37.0, −2.9]	34.6 [21.5, 47.8]	−0.32 [−0.38, −0.26]
	2	−49.1 [−84.3, −13.9]	39.6 [12.5, 66.7]	−0.51 [−0.64, −0.39]
	3+	−106.5 [−192.3, −20.7]	29.2 [−36.9, 95.3]	−0.78 [−1.09, −0.47]
**Total previous births**	0	Ref	Ref	Ref
	1	136.6 [118.2, 155.0]	80.7 [66.5, 94.9]	0.28 [0.21, 0.34]
	2+	178.0 [124.5, 231.4]	99.1 [57.9, 140.3]	0.39 [0.20, 0.59]
**Funding**	NHS	Ref	Ref	Ref
	Private	13.6 [3.9, 23.3]	23.2 [15.7, 30.7]	−0.07 [−0.10, −0.03]
	Not Recorded	5.6 [−29.7, 40.9]	29.2 [2.0, 56.3]	−0.15 [−0.28, −0.03]
**Country**	England	Ref	Ref	Ref
	N Ireland	64.2 [37.4, 90.9]	84.8 [64.1, 105.4]	−0.13 [−0.23, −0.04]
	Scotland	51.9 [22.7, 81.2]	40.8 [18.3, 63.4]	0.10 [−0.01, 0.20]
	Wales	−20.3 [−80.5, 40.0]	5.2 [−41.3, 51.6]	−0.13 [−0.35, 0.08]
	Missing	127.0 [−697.3, 951.4]	32.2 [−603.0, 667.4]	0.43 [−2.54, 3.41]
**Ovulatory infertility**	No	Ref	Ref	Ref
	Yes	−30.2 [−40.4, −20.1]	−5.9 [−13.7, 1.9]	−0.15 [−0.18, −0.11]
**Tubal infertility**	No	Ref	Ref	Ref
	Yes	−14.9 [−25.0, −4.8]	3.5 [−4.3, 11.3]	−0.11 [−0.15, −0.07]

(continued)

Supplementary Table S1. (continued)

		Raw BW (g)	gaBW (g)[1]	Gestation (weeks)
**Endometriosis**	No	Ref	Ref	Ref
	Yes	−21.3 [−34.1, −8.5]	−13.4 [−23.2, −3.5]	−0.04 [−0.09, 0.00]
**Male infertility**	No	Ref	Ref	Ref
	Yes	16.3 [7.5, 25.2]	1.2 [−5.6, 8.0]	0.09 [0.06, 0.13]
**Unknown infertility cause**	No	Ref	Ref	Ref
	Yes	3.5 [−6.00, 13.0]	−7.5 [−14.8, −0.5]	0.07 [0.03, 0.10]
**Embryos transferred**	Missing	−50.0 [−141.5, 41.4]	−57.4 [−127.9, 13.1]	−0.02 [−0.35, 0.31]
	1	12.1 [3.00, 21.2]	6.1 [−1.0, 13.1]	0.03 [−0.01, 0.06]
	2	Ref	Ref	Ref
	3	−6.2 [−18.9, 6.5]	5.4 [−4.4, 15.2]	−0.07 [−0.11, −0.02]

Models also included for treatment centre and year as categorical variables.

[1] Gestation adjusted birthweight fitted using a cubic function.

© The Author(s) 2026. Published by Oxford University Press on behalf of European Society of Human Reproduction and Embryology.
This is an Open Access article distributed under the terms of the Creative Commons Attribution License (https://creativecommons.org/licenses/by/4.0/), which permits unrestricted reuse, distribution, and reproduction in any medium, provided the original work is properly cited.
Human Reproduction, 2026, 00, 1–15
https://doi.org/10.1093/humrep/deag140
Original Article
